# CD44 standard and CD44v10 isoform expression on leukemia cells distinctly influences niche embedding of hematopoietic stem cells

**DOI:** 10.1186/1756-8722-7-29

**Published:** 2014-03-31

**Authors:** Ulrike Erb, Amelie Pajip Megaptche, Xiaoyu Gu, Markus W Büchler, Margot Zöller

**Affiliations:** 1 Department of Tumor Cell Biology, University Hospital of Surgery, Heidelberg, Germany; 2 University Hospital of Surgery, Heidelberg, Germany

**Keywords:** Leukemia, Antibody therapy, CD44v10, Hematopoiesis, Bone marrow stroma

## Abstract

**Background:**

A blockade of CD44 is considered a therapeutic option for the elimination of leukemia initiating cells. However, anti-panCD44 can interfere with hematopoiesis. Therefore we explored, whether a CD44 variant isoform (CD44v)-specific antibody can inhibit leukemia growth without attacking hematopoiesis. As a model we used CD44v10 transfected EL4 thymoma cells (EL4-v10).

**Methods:**

The therapeutic efficacy of anti-panCD44 and anti-CD44v10 was evaluated after intravenous application of EL4/EL4-v10. Ex vivo and in vitro studies evaluated the impact of anti-panCD44 and anti-CD44v10 as well as of EL4 and EL4-v10 on hematopoietic stem cells (HSC) in cocultures with bone marrow stroma cells with a focus on adhesion, migration, cell cycle progression and apoptosis resistance.

**Results:**

Intravenously injected EL4-v10 grow in bone marrow and spleen. Anti-panCD44 and, more pronounced anti-CD44v10 prolong the survival time. The higher efficacy of anti-CD44v10 compared to anti-panCD44 does not rely on stronger antibody-dependent cellular cytotoxicity or on promoting EL4-v10 apoptosis. Instead, EL4 compete with HSC niche embedding. This has consequences on quiescence and apoptosis-protecting signals provided by the stroma. Anti-panCD44, too, more efficiently affected embedding of HSC than of EL4 in the bone marrow stroma. EL4-v10, by catching osteopontin, migrated on bone marrow stroma and did not or weakly interfere with HSC adhesion. Anti-CD44v10, too, did not affect the HSC – bone marrow stroma crosstalk.

**Conclusion:**

The therapeutic effect of anti-panCD44 and anti-CD44v10 is based on stimulation of antibody-dependent cellular cytotoxicity. The superiority of anti-CD44v10 is partly due to blocking CD44v10-stimulated osteopontin expression that could drive HSC out of the niche. However, the main reason for the superiority of anti-CD44v10 relies on neither EL4-v10 nor anti-CD44v10 severely interfering with HSC – stroma cell interactions that, on the other hand, are affected by EL4 and anti-panCD44. Anti-panCD44 disturbing HSC embedding in the osteogenic niche weakens its therapeutic effect towards EL4. Thus, as far as leukemic cells express CD44v isoforms, the therapeutic use of anti-panCD44 should be avoided in favor of CD44v-specific antibodies.

## Background

**Figure 1:**
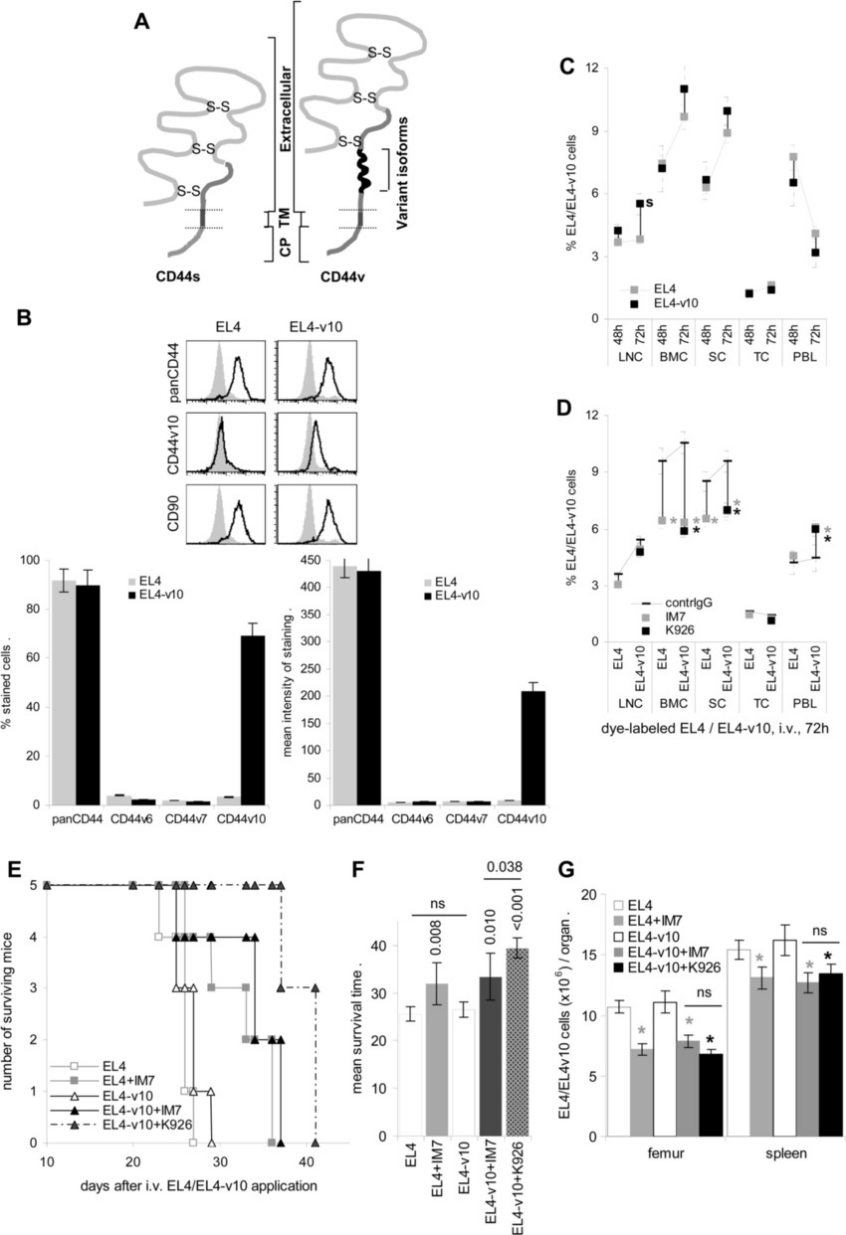
**Impact of anti-CD44 on intra-marrow growth of EL4 and EL4-v10. (A)** CD44 is composed of a cytoplasmic domain (CP), a transmembrane domain (TM), and an extracellular domain that is composed of a stalk like and a globular region. The HA binding site is located in the globular region. Variant exon products are inserted in the stalk like region. **(B)** CD44 and CD44v10 expression in EL4 and EL4-v10 cells: flow cytometry, mean ± SD of % stained cells and intensity of staining (triplicates) and example. **(C,D)** Homing of i.v. injected CFSE-labeled EL4/EL4-v10 (1×10^7^) was evaluated after 48 h and 72 h in dispersed lymphatic organs by flow cytometry, mean ± SD of the % CFSE-labeled tumor cells/organ (3 mice/group); **(B)** significant differences (p <0.05) between EL4 versus EL4-v10: s; **(D)** significant inhibition (p <0.05) by concomitant i.v. antibody (100 μg/mouse) application: *. **(E,F)** Mice received an i.v. injection of 1×10^6^ EL4/EL4-v10 and 2×/week 100 μg IM7 or K926; the first injection following 1 h after tumor cell application; **(E)** survival time and rate of 5 mice/group; **(F)** survival time (mean ± SD), significant differences by IM7 or K926 as well as differences between EL4 versus EL4-v10 (ns: not significant) and between IM7 versus K926 in EL4-v10 bearing mice (p 0.038) are indicated. **(G)** Recovery of tumor cells in femur and spleen 2 wk after tumor cell application was evaluated by flow cytometry; the mean number ± SD of 3 mice/group is shown; significant differences (p <0.05) by IM7 or K926 application: *; differences between IM7 and K926 were not significant (ns). EL4 and EL4-v10 preferentially home into BM and spleen. Anti-panCD44 (IM7) and anti-CD44v10 (K926) prolong the survival time, with K926 showing higher efficacy in EL4-v10 inoculated mice.

The adhesion molecule CD44 is a glycoprotein that varies in size due to N- and O-glycosylation and insertion of alternatively spliced variable exon products (Figure 1A) [1-4]. It is expressed on many cells, particularly hematopoietic cells including hematopoietic stem cells (HSC), but also leukemia-initiating cells (LIC) [5-9], where CD44 attracted considerable interest as a functionally important HSC and LIC marker [5,10,11].

CD44 is important for HSC and LIC homing [12-14]. Anti-CD44 prohibits the development of cobblestone areas in murine long term bone marrow cultures (LTBMC) [15,16]. Bone marrow stroma formation also requires CD44 that supports the process by induction of IL-6 secretion [17]. HSC synthesize and express hyaluronic acid (HA) and HA expression correlates with selective migration of HSC to the endosteal niche [18]. Furthermore, anti-CD44 efficiently mobilizes HSC [16,19,20]. HSC homing can be blocked by anti-CD44, soluble HA or hyaluronidase treatment [21]. In addition, CXCL12 stimulates adhesion of progenitor cells via CD44, which demonstrates a cross-talk between CD44 and CXCR4 signaling and suggests a key role of HA and CD44 in CXCL12-dependent transendothelial migration of HSC and their anchorage within specific niches [19]. Besides contributing to homing and settlement of HSC in the bone marrow niche, CD44 promotes quiescence versus proliferation [22-24].

CD44 also accounts for LIC homing [24]. In acute myeloid leukemia (AML) CD44 is required for the transport to the stem cell-supportive osteogenic niche and anti-CD44 alters the fate of AML-IC by inducing differentiation [25]. In a mouse model of chronic myeloid leukemia (CML), BCR-ABL1-transduced progenitors from CD44-mutant donors show defects in bone marrow homing. This results in decreased engraftment and impaired CML-like disease induction, pointing towards LIC being more dependent on CD44 for homing than HSC [26]. However, a marker/signal transduction molecule that is shared by LIC and HSC presents a risk factor, when trying to attack the LIC [5,27]. Our studies, confirming that leukemia cell homing and growth is retarded by a CD44-specific antibody, pointed towards an antibody blockade of CD44 during reconstitution to affect HSC embedding more severely than leukemia growth [28,29].

Based on the finding that leukemia cells efficiently compete with HSC under the condition of myeloablation and reconstitution [28,29], we here asked whether anti-CD44 also interferes with hematopoiesis in the non-compromised leukemia-bearing host and whether such interference could be prevented by replacing anti-panCD44 with a CD44 variant isoform-specific antibody. We choose the CD44v10 cDNA transfected thymoma EL4 (EL4-v10). CD44v10 is expressed on subpopulations of macrophages (Mϕ), activated Th1 cells and hematopoietic progenitor cells, particularly of the B cell lineage [30-34]. Upregulated CD44v10 expression correlates with Hodgkin’s disease relapse [34] and in multiple myeloma it supports adhesion to marrow endothelial cells [35]. Upregulated CD44v10 expression, though not reaching statistical significance, was also observed in some patients with AML [36]. CD44v10 appeared particularly suited as it is very weakly expressed on HSC, but is a receptor for osteopontin (OPN), that according to reports of several groups should support motility of hematopoietic stem and progenitor cells [37,38]. Thus, we expected a blockade of CD44v10 not or hardly to affect HSC.

We report that anti-CD44v10 displays higher efficacy than anti-panCD44 in retarding EL4-v10 growth. This is an indirect effect, as anti-CD44v10, as expected, distinct to anti-panCD44, does not disturb hematopoiesis. In addition, CD44v10 being an OPN ligand, EL4-v10 migrate on bone marrow stroma (BM-Str), but do not tightly adhere, leaving space for HSC settlement.

## Results

The interaction with the osteogenic niche is important for maintenance of HSC quiescence and timely maturation [39]. One of the molecules of central importance in these processes is CD44 [5,10], which also becomes apparent by anti-CD44 efficiently mobilizing HSC [20]. CD44 also is frequently overexpressed in leukemia such that LIC can be driven into differentiation and death by anti-CD44 [7,25,26]. Thus, we asked whether by replacing anti-panCD44 by a CD44 variant isoform-specific antibody, anti-leukemic effects can be maintained without interfering with hematopoiesis using as a model highly standard CD44 (CD44s)^+^ EL4, which do not express CD44v isoforms (Zöller, unpublished) and were transfected with CD44v10 (EL4-v10) (Figure 1B).

### The impact of CD44v10 and anti-CD44v10 on bone marrow homing and intra-marrow growth

Mice received an intravenous (i.v.) injection of 1×10^7^ CFSE-labeled EL4 or EL4-v10 concomitantly with control IgG, anti-panCD44 (IM7) or anti-CD44v10 (K926). Mice were sacrificed after 48 h and 72 h, hematopoietic organs were excised and the recovery of fluorescent EL4 and EL4-v10 was evaluated by flow cytometry. Both, EL4 and EL4-v10 preferentially home into bone marrow (BM) and spleen with EL4-v10 having a slight advantage in settlement in lymph nodes (LN) (Figure 1C). IM7 and K926 interfere with homing into BM and spleen, which is accompanied by a pronounced retention in the peripheral blood. IM7 and K926 displayed comparable efficacy in inhibiting EL4 and EL4-v10 homing (Figure 1D).

Mice receiving i.v. injections of 1×10^6^ EL4/EL4-v10 became moribund after 27d to 29d. The mean survival time was prolonged to 32d and 33d, respectively, by IM7 treatment. Instead, EL4-v10-bearing mice receiving K926 had a mean survival time of 38.5d, which differed significantly from the survival time of EL4-v10 bearing mice receiving IM7 (Figure 1E and F). When mice were sacrificed after 21d, comparable numbers of EL4 and EL4v10 cells were recovered in the BM and the spleen, respectively. IM7 and K926 had a minor impact on the recovery in the spleen, but a significantly reduced number was seen in the BM. Distinct to the superior impact of K926 on the survival time, recovery of EL4-v10 in the BM of K926-treated mice did not significantly differ from that in IM7-treated mice (Figure 1G).

Taken together, EL4 and EL4-v10 preferentially home into BM and spleen. There is no evidence for increased tumorigenicity by CD44v10 expression. A blockade of CD44 retards homing and leukemia growth in BM and spleen. In EL4-v10 inoculated mice a blockade of CD44v10 with K926 is more efficient.

### IM7 and K926 promote antibody-dependent cellular cytotoxicity, IM7 interferes with hematopoiesis

**Figure 2:**
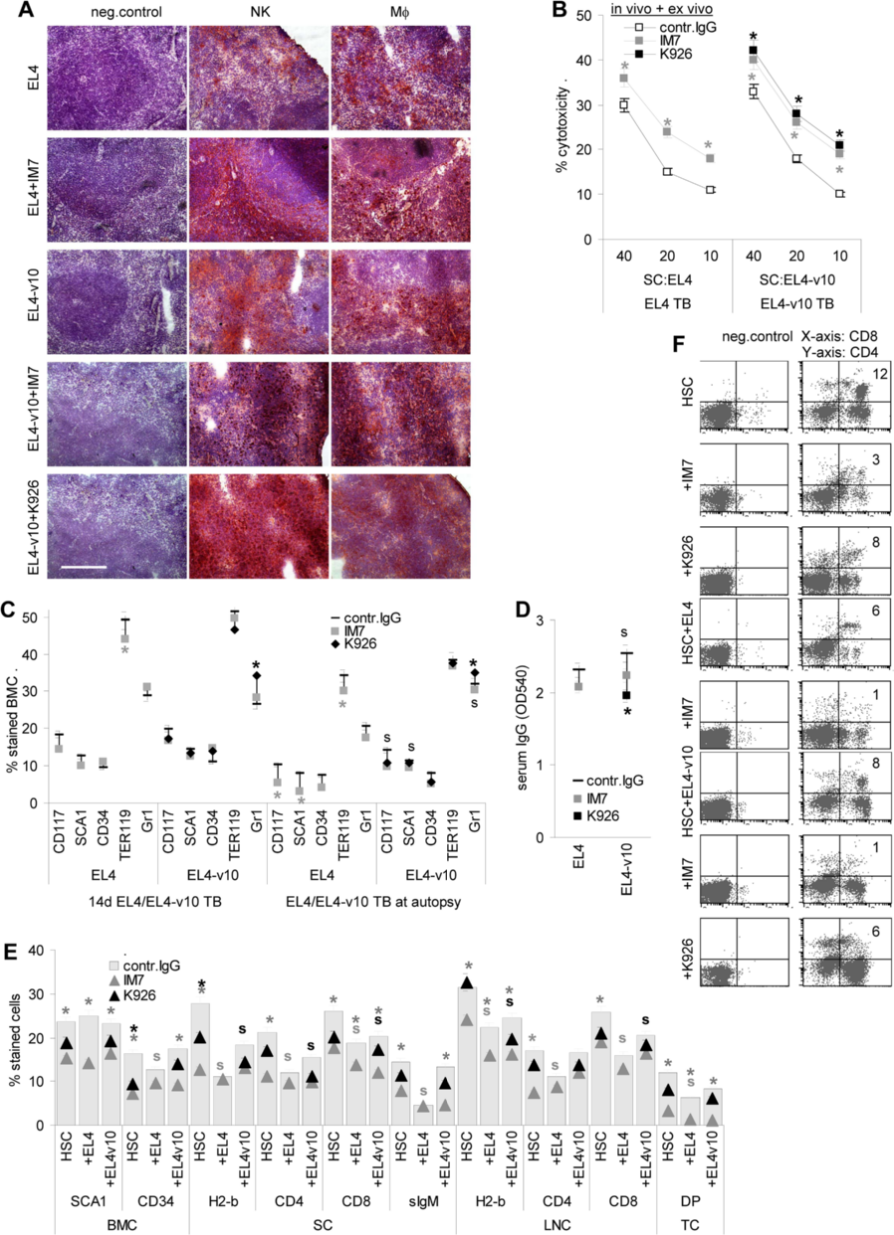
**Effector leukocytes and antibody-promoted ADCC in EL4 and EL4-v10 bearing mice. (A)** Recovery of NK and Mf by immunohistochemistry (cryosections of EL4 and EL4-v10 TB spleen): hematoxilin (blue) staining of nuclei, antibodies recognizing NK cells or Mf (red); scale bar: 100 μm. **(B)** ADCC in 3 wk EL4 and EL4-v10 TB SC after 2d in RPMI1640/100 U/ml IL2 and 10 μg/ml IM7 or K926 corresponding to in vivo treatment; mean±SD (triplicates); significant differences (p <0.05) by antibody treatment: *. **(C)** Recovery of HSC and progenitors in 2 wk TB and at autopsy (flow cytometry); mean±SD (3 mice/group); **(D)** serum IgG recovery (ELISA) in 2 wk TB, OD405 at 1:20 serum dilution (mean±SD, 3 mice/group); **(C,D)** significant differences (p <0.05) between EL4 and EL4-v10 TB: s; and by IM7 or K926 treatment: *. **(E,F)** BALB/c SCID mice (5/group) received 2×106 HSC or HSC plus 1×106 EL4 or EL4-v10 (i.v.) and 100 μg/mouse IM7 or K926 (i.v.) 24 h after HSC application. Antibody application was repeated in 5d intervals. BM, thymus, spleen and LN were excised after 3 wk for recovery of the indicated cell populations by flow cytometry. **(E)** Mean percent±SD/organ (5 mice/group) and **(F)** representative examples; significant differences (p <0.05) between tumor-free and EL4 or EL4-v10 TB: s and by IM7 or K926 treatment: *. NK and Mf are enriched in splenic tumors in IM7- or K926-treated mice, which corresponds to increased ADCC. Significantly fewer HSC are recovered in EL4 than EL4-v10 TB, HSC being further decreased in IM7-treated EL4 TB. There is a slight increase in Gr1+ cells in the BM and a decrease in serum IgG in K926-treated EL4-v10 TB. Upon transfer of HSC into SCID mice, maturation/differentiation into lymphocytes is severely impaired in EL4 TB and is most strongly reduced by concomitant IM7 application. K926 slightly affected B cell recovery.

Spleen sections of EL4 and EL4v10 tumor-bearer (TB) revealed recruitment particularly of NK and macrophages (Mϕ) in IM7- and K926-treated mice without significant differences between EL4 versus EL4v10 or between IM7 versus K926 treatment (Figure 2A). Antibody-dependent cellular cytotoxicity (ADCC) of IM7- or K926-treated 2 wk EL4-and EL4-v10 TB did not significantly differ (Figure 2B).

Taken together, antibody treatment promotes leukocyte recruitment into tumor nodules and suffices for strong ADCC, which does not differ between IM7 versus K926 treatment, excluding that effector cell activation accounts for the prolonged survival time of K926-treated EL4-v10 TB. Thus, we asked for the impact of EL4 versus EL4v10 as well as of IM7 versus K926 on hematopoiesis.

When controlling for the presence of HSC, we noted a slight decrease in cells expressing the HSC markers CD117 and SCA1 in the BM of IM7-treated early TB, which became significant in late TB. In addition, fewer HSC were recovered from late EL4 than EL4-v10 TB (Figure 2C). In late EL4-v10 TB a minor retention of Gr1^+^ cells in the BM and a slight reduction in serum IgG was noted (Figure 2C and D), which is in line with CD44v10 being engaged in monocyte and B cell maturation [30]. Reduced recovery of HSC in IM7-treated late TB could be in line with IM7, distinct to K926, interfering with HSC embedding in the osteogenic niche. The higher recovery of HSC in late EL4-v10 than EL4 TB might be indicative for EL4-v10 not or less efficiently competing with HSC for stroma adhesion.

To consolidate these findings, BALB/c SCID mice were concomitantly "reconstituted" with C57BL6 HSC and EL4 or EL4-v10 and received weekly injections of IM7 or K926. According to our hypothesis, T- and B-cell recovery should be impaired in EL4 versus EL4-v10 bearing mice and should be additionally affected by IM7, but not K926. Three weeks after i.v. HSC and EL4/EL4-v10 application, fewer donor cells were recovered in spleen and lymph nodes of TB than control mice, the decrease was more pronounced in EL4 than EL4-v10 TB. IM7 and not or less pronounced K926 affected the recovery of donor cells also in control mice. Furthermore, the recovery of CD4^+^ and CD8^+^ cells was more strongly reduced in lymph nodes and spleen of EL4 than EL4-v10 TB and hardly any B cells were recovered in the spleen of EL4 TB. Only IM7 significantly affected the lymphocyte recovery, which also accounted for SCID mice only receiving HSC. Recovery of CD4^+^CD8^+^ TC also differed between TB and tumor-free mice and recovery of double-positive TC was strikingly reduced in IM7-treated mice. Likely due to the contribution of the SCID mice derived HSC, SCA1^+^ cells were not strikingly affected by EL4 or EL4-v10, but were reduced in IM7-treated mice. Recovery of CD34^+^ cells was reduced in EL4, but not EL4-v10 TB and IM7 contributed to a reduction in tumor-free and EL4-v10 TB mice (Figure 2E and F).

The strongly reduced recovery of mature lymphocyte in reconstituted EL4 TB mice confirming the pronounced impact of EL4 on hematopoiesis, we aimed to answer the question, why EL4-v10 and K926 exert a weaker effect on hematopoiesis and explored the interaction of EL4, EL4-v10 and HSC with BM-Str in dependence on the presence of IM7 or K926.

### Crosstalk between EL4/EL4-v10 and bone marrow stroma

**Figure 3:**
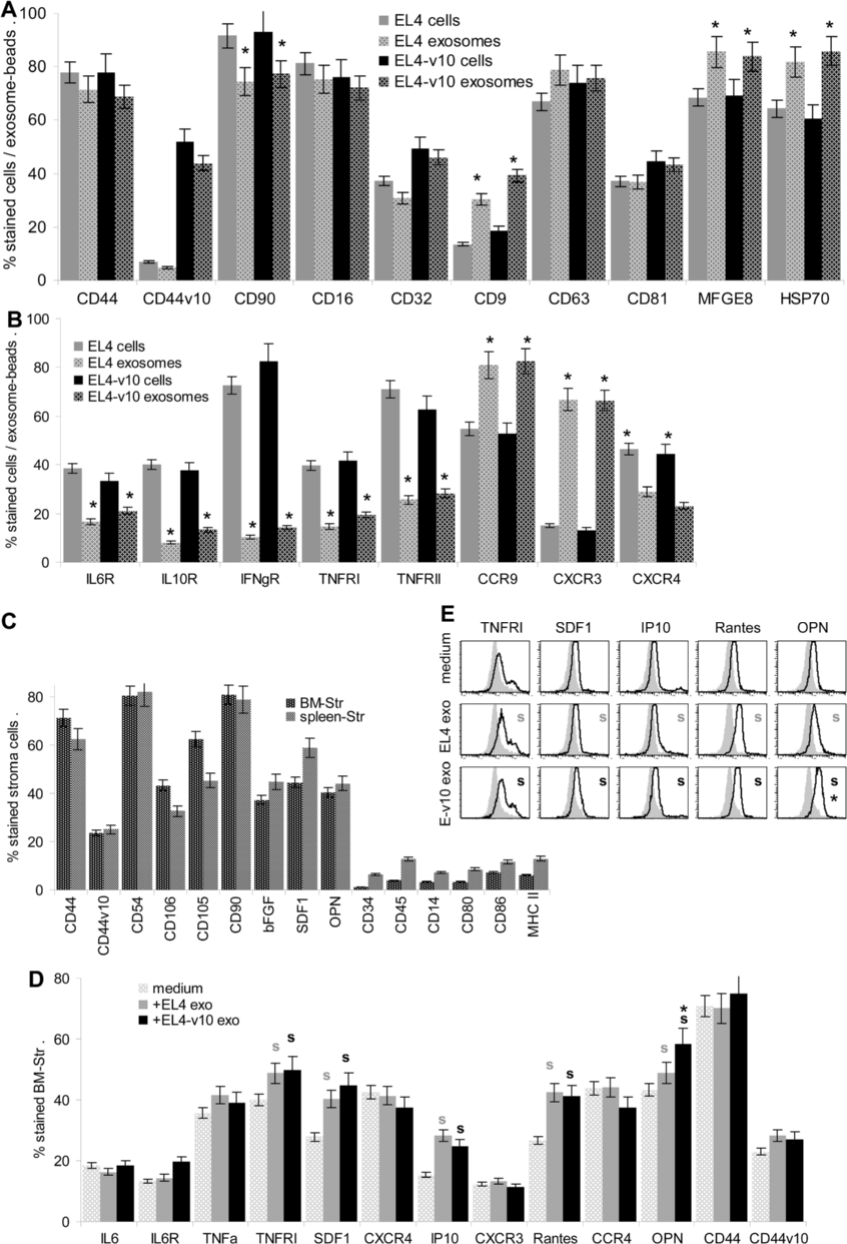
**Impact of EL4 and EL4-v10 exosomes on stroma cells. (A,B)** Flow-cytometry of EL4 and EL4-v10 cells and exosomes; mean percent stained cells ± SD (triplicates) and representative example; significant differences between cells versus exosomes: *. **(C)** Flow cytometry of BM-Str and spleen-Str and **(D,E)** of BM-Str treated for 24 h with EL4 and EL4-v10 exosomes; mean ± SD percent stained Str (triplicates) and representative examples; significant changes (p <0.05) by coculture with EL4 or EL4-v10 exo: s. Experiments **A**-**E** were repeated 3-times. BM- and spleen-Str are highly CD44^+^ and express stroma cell markers, there was no contamination by hematopoietic cells. EL4 and EL4-v10 exosomes promoted upregulation of TNFRI and of the chemokines SDF1, IP10, Rantes and OPN, only expression of the latter becoming more strongly induced by EL4-v10 than EL4.

In advance of evaluating the crosstalk between BM-Str or spleen-Str and EL4/EL4-v10, we controlled, whether EL4 and EL4-v10 affect the stroma. This was done by culturing EL4 and EL4-v10 exosomes with stroma cells to avoid a feedback towards EL4/EL4-v10 tumor cells by stroma cells. Adhesion molecule expression in EL4/EL4-v10 exosomes resembles that in cells. Expression of the tetraspanin CD9 as well as of MFGE8 and HSP70 is higher than in cells (Figure 3A). Cytokine and chemokine recovery was lower than in cells (data not shown). Cytokine receptors also were recovered at a lower level in exosomes, but CCR9 and CXCR3 were upregulated (Figure 3B). Notably, besides CD44v10 expression we did not observe major differences between EL4 versus EL4-v10 exosomes.

BM- and spleen-Str highly express CD44 CD54, CD90 and CD105, they are partly CD106 and weakly CD44v10 positive and express bFGF, SDF1 and OPN. Contamination by hematopoietic cells was excluded by the absence or very low expression of CD34, CD45, CD14, CD80, CD86 and MHC II particularly in BM-Str (Figure 3C). EL4 and EL4-v10 exosomes promoted upregulation of Rantes, IP10, SDF1 and OPN on BM-Str. EL4 and EL4-v10 exosomes promoted TNFRI upregulation, expression of other chemokine receptors was not affected (Figure 3D and data not shown). It is important to note that with the exception of OPN no significant differences were seen concerning the impact of EL4 versus EL4-v10 exosomes on BM-Str, which allowed to judge on the crosstalk between stroma and EL4 versus EL4-v10.

**Figure 4:**
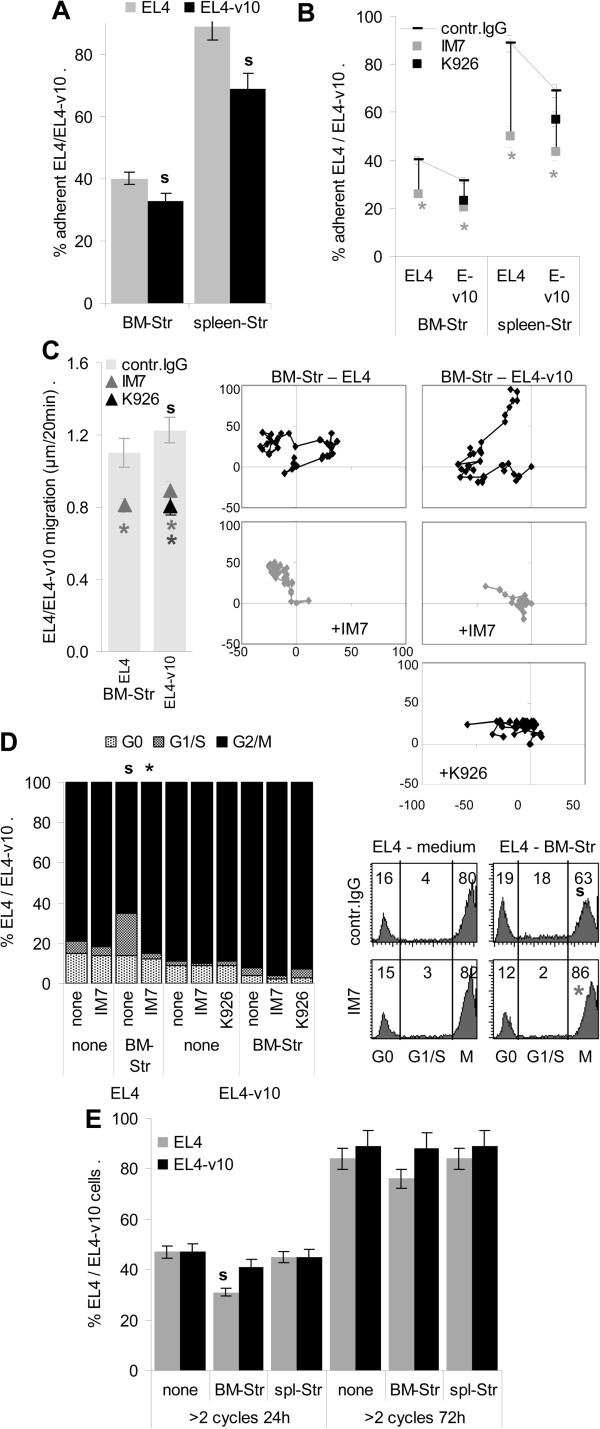
**Crosstalk of EL4, EL4-v10 with bone marrow- and spleen-stroma. (A,B)** CFSE-labeled EL4 and EL4-v10 (1×105) were seeded in 24-well plates on a confluent BM-Str monolayer, cultures containing 10 μg/ml IM7 or K926. After 4 h (37°C) and washing, cells were lysed to determine fluorescence. Adhesion of EL4 and EL4-v10 to BM-Str and spleen-Str and inhibition by antibodies (mean±SD, triplicates); **(A)** significant differences (p <0.05) in adhesion of EL4 vs. EL4-v10: s; and **(B)** by antibody: *. **(C)** CFSE-labeled EL4 and EL4-v10 (1×105) were seeded in 24-well plates on a 90% confluent BM-Str monolayer, cultures containing 10 μg/ml IM7 or K926. Migration of EL4 and EL4-v10 on BM-Str (videomicroscopy, pictures every 20 min for 12 h). Mean±SD of migration (20 EL4 or EL4-v10/well) and representative examples; significant differences (p <0.05) between EL4 vs. EL4-v10: s, and in the presence of antibody: *; **(D)** EL4 and EL4-v10 (2×105) were seeded in triplicates in 24-well plates coated with BSA or a BM-Str monolayer. After starving (48 h) RPMI1640/10% FCS was added (2 h), medium contained 10 μg/ml IM7 or K926. After washing and fixation (ethanol) EL4/EL4-v10 were stained with PI; Mean values (triplicates) of cell cycle progression and representative examples; significant differences (p <0.05) in dependence on BM-Str: s, and in the presence of antibody: *; **(E)** CFSE-labeled EL4/EL4-v10 (2×105) were seeded in 24-well plates coated with BSA or BM-Str or spleen-Str. Proliferation of EL4 and EL4-v10 was evaluated by flow cytometry (CFSE dilution) after 24 h and 72 h; mean±SD (triplicates) of EL4/EL4-v10 having progressed through 2 divisions; significant differences (p <0.05) in the presence of stroma: s. Experiments **A**-**E** were repeated 3-times. EL4 adhere slightly better than EL4-v10 to BM- and spleen-Str, adhesion is mostly inhibited by IM7. Contact with BM-Str affects EL4 cell cycle progression, which is neutralized by IM7.

EL4 adhere more strongly to BM- and spleen-Str than EL4-v10 and adhesion is significantly inhibited only by IM7 (Figure 4A and 4B). Distinct to reduced adhesion, EL4-v10 more readily than EL4 migrated on BM-Str. Migration was inhibited by IM7 as well as K926 (Figure 4C).

**Figure 5:**
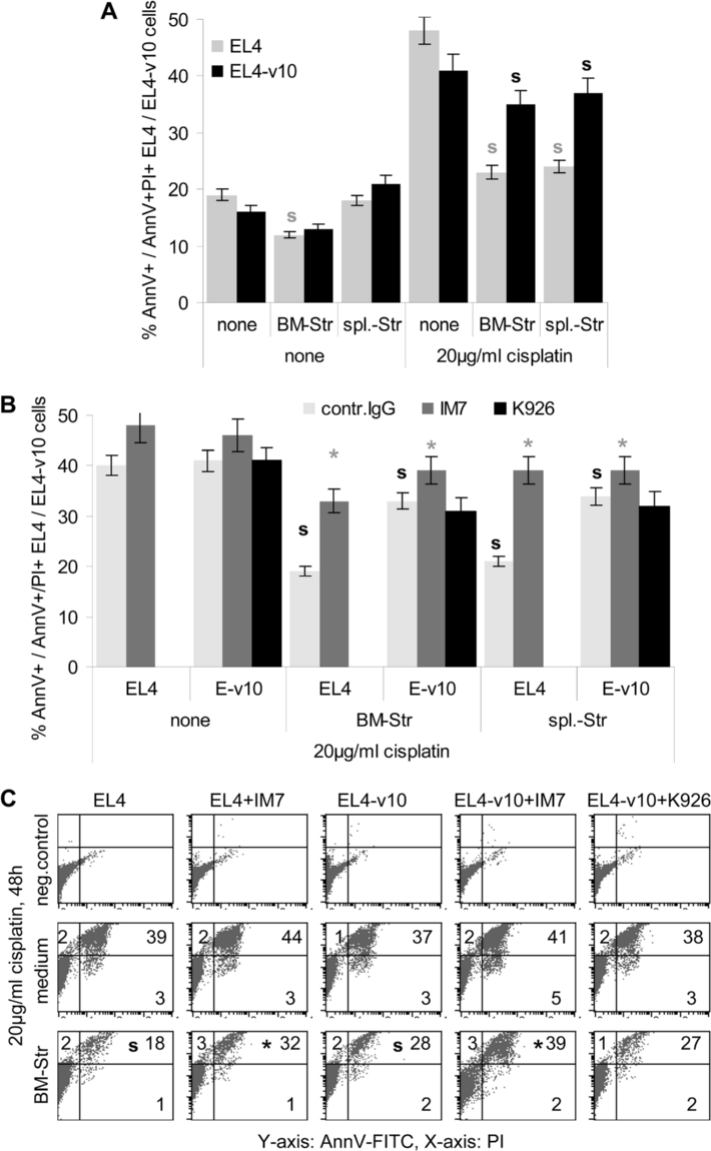
**Stroma-promoted apoptosis protection of EL4, EL4-v10. (A-C)** EL4/EL4-v10 (5×10^5^) were seeded in triplicates on BSA or BM-Str or spleen-Str coated 24-well plates. Cell were cultured for 48 h in the presence of 20 μg/ml cisplatin; where indicated cultures contained 10 μg/ml IM7 or K926. EL4/EL4-v10 were thoroughly collected and apoptotic EL4 / EL4-v10 (AnnV^+^ or AnnV^+^/PI^+^) were determined by flow cytometry; **(A,B)** mean ± SD (triplicates) of % AnnV^+^ and AnnV^+^/PI^+^ cells and **(C)** representative examples; significant differences (p <0.05) in the presence of stroma: s, significant differences (p <0.05) in the presence of IM7 or K926: *. The experiment was repeated 3-times. BM- and spleen-Str exert a strong apoptosis protecting effect on EL4 and EL4-v10 that is neutralized by IM7, but not by K926.

BM-Str also distinctly affected EL4 and EL4-v10 cell cycle progression such that BM-Str slightly inhibited EL4, but not EL4-v10 cell cycle progression. Cell cycle retardation was neutralized by IM7 (Figure 4D). In line with retarded cell cycle progression, BM-Str also influenced EL4, but not EL4-v10 proliferation. However, the impact of BM-Str was weak and was no longer significant after 72 h of coculture (Figure 4E). Finally, BM- and spleen-Str protected EL4 and, less efficiently EL4-v10 from cisplatin-induced apoptosis (Figure 5A and C). IM7 and K926 did not significantly affect low apoptosis in the absence of cisplatin. Instead in the presence of cisplatin, the protective effect of BM- and spleen-Str was waved by IM7, whereas K926 exerted no significant effect (Figure 5B and C).

These findings indicate a more intense crosstalk between stroma cells and EL4 than EL4-v10, which affects adhesion, cell cycle progression and apoptosis protection. This crosstalk mostly is CD44s-dependent, as - with the exception of migration - it is efficiently blocked by IM7, but hardly by K926. According to these findings, one would expect a stronger effect of IM7 than of K926 on EL4/EL4-v10 growth in the bone marrow. As we observed the opposite, the suspicion arose that IM7 affects hematopoiesis. Alternatively, though not mutually exclusive, EL4 more efficiently compete for the endosteal niche, thereby negating the IM7 anti-leukemic effect.

### The impact of EL4/EL4-v10 on the crosstalk between HSC and bone marrow stroma

Experiments controlling for the competition between HSC and EL4/EL4-v10 with BM-Str as well as for the impact of IM7 and K926 were performed with mitomycinC-treated EL4/EL4-v10 cells or exosomes. As we did not observe major differences between exosomes and cells, for clarity of presentation experiments shown were mostly performed with exosomes.

**Figure 6:**
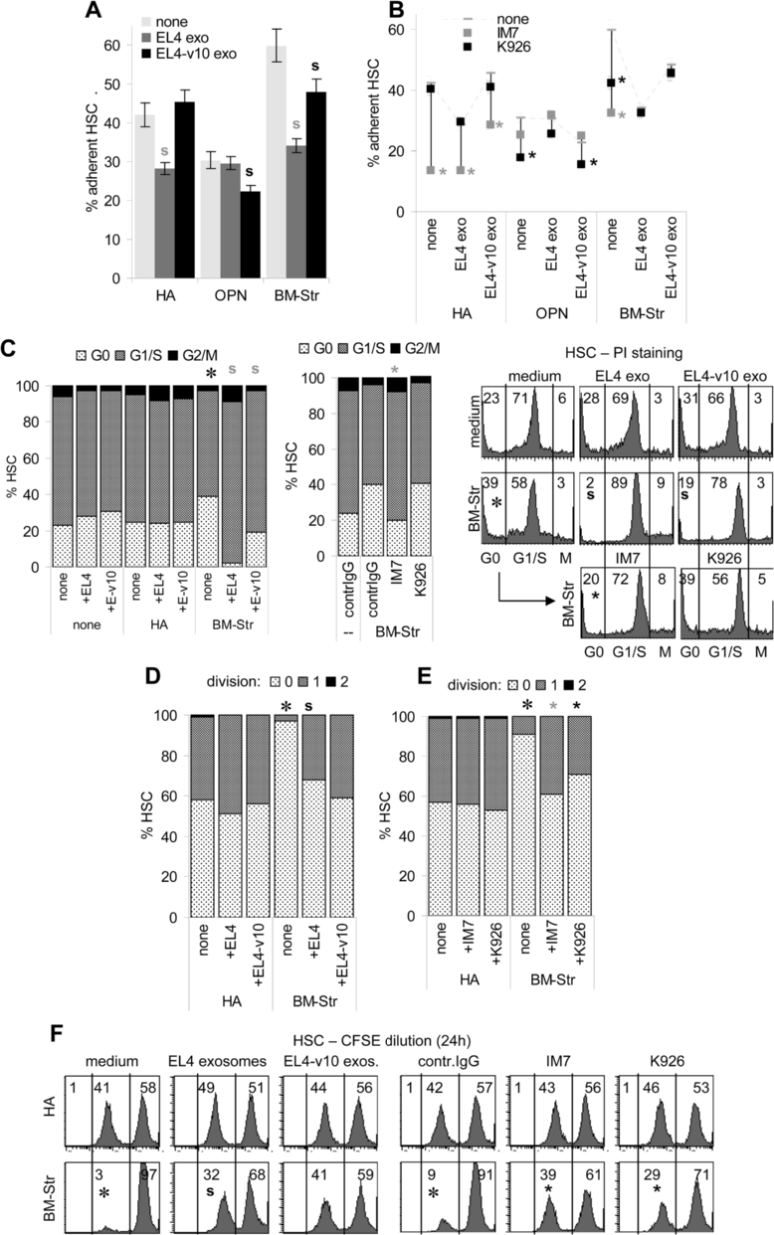
**Impact of EL4 and EL4-v10 on bone marrow stroma-promoted HSC quiescence. (A,B)** CFSE-labeled HSC (1×10^6^) were cocultured with EL4 or EL4-v10 exosomes (10 μg/ml) on HA, OPN or BM-Str-coated plates for 4 h. After removing non-adherent cells, cells were lysed and fluorescence was determined photometrically. **(A)** Percent HSC (mean ± SD, triplicates) adhering to HA, OPN or BM-Str in dependence on EL4/EL4-v10 exosomes; significant differences (p <0.05) by EL4 or EL4-v10: s; **(B)** percent HSC (mean ± SD, triplicates) adhering to HA, OPN or BM-Str in dependence on IM7 or K926; significant (p <0.05) antibody inhibition: *; **(C)** HSC (1×10^6^) were seeded on BSA, HA or BM-Str coated 24-well plates and starved for 24 h. Thereafter HSC were cultured for 2 h in medium containing 10% FCS; where indicated cultures contained EL4/EL4-v10 exosomes (10 μg/ml) or IM7 or K926 (10 μg/ml). HSC were collected and treated as described in Figure 4D; PI staining was evaluated by flow cytometry; the mean percent (triplicates) of cells in G0, G1/S and G2/M and representative examples are shown; significant impact (p <0.05) of BM-Str: *; significant differences (p <0.05) by EL4 or EL4-v10: s, significant differences (p <0.05) by antibody: *; **(D-F)** CFSE-labeled HSC (1×10^6^) were cultured on HA or BM-Str coated 24-well plates for 24 h; cultures contained EL4 or EL4-v10 exosomes (10 μg/ml) or IM7 or K926 (10 μg/ml). HSC were collected and proliferation was evaluated by CFSE dilution (flow-cytometry), the mean% (triplicates) of HSC that had not divided or divided 1-times or 2-times and representative examples are shown; significant differences (p <0.05) by BM-Str as compared to HA: *, significant differences (p <0.05) by EL4 / EL4-v10 exosomes: s, significant differences (p <0.05) by antibody: *. Experiments were repeated 3-times. EL4, more pronounced than EL4-v10 interfere with HSC – BM-Str adhesion and HSC quiescence, which is also disturbed by IM7.

When CFSE-labeled HSC were seeded on HA-, OPN- or BM-Str-coated plates, adhesion to HA and BM-Str was more strongly impaired in the presence of EL4 than EL4-v10, whereas adhesion to OPN was mostly inhibited by EL4-v10 (Figure 6A). IM7 blocked adhesion of HSC to HA and BM-Str; K926 blocked adhesion to OPN and, though less efficiently, adhesion to BM-Str. Reduced HSC adhesion to HA in the presence of EL4 becomes further impaired by IM7, reduced adhesion to OPN in the presence of EL4-v10 was only affected by K926. Instead, weaker adhesion of HSC to BM-Str in the presence of EL4/EL4-v10 was neither affected by IM7 nor K926 (Figure 6B). EL4/EL4-v10 competing with HSC for binding, but HSC lodging not being further impaired by IM7, could be due to EL4/EL4-v10 efficiently catching IM7.

**Figure 7:**
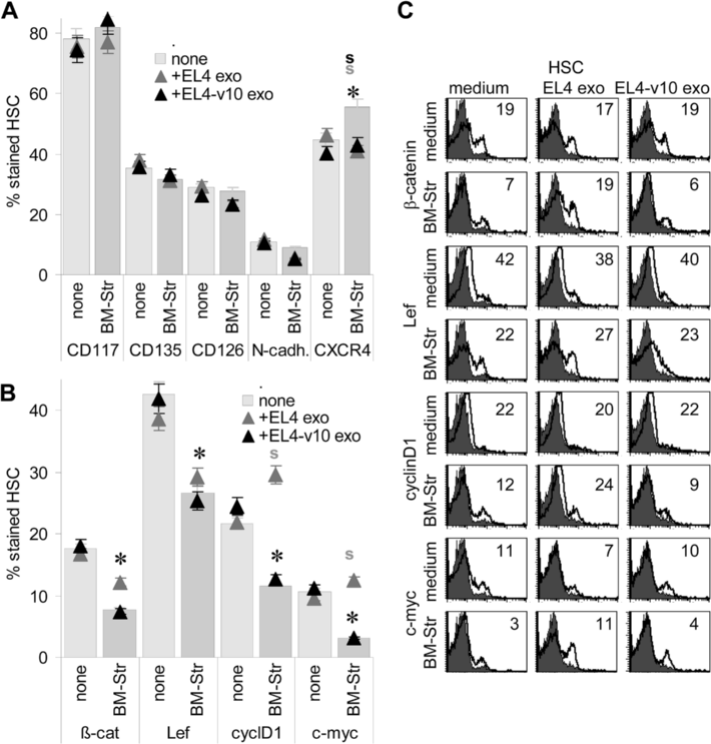
**Impact of EL4 and EL4-v10 on HSC marker and cell cycle-related protein expression.** HSC (1×10^6^) were cultured for 24 h on BSA or BM-Str coated 24-well plates; where indicated, cultures contained EL4 or EL4-v10 exosomes. HSC were collected and expression of **(A)** HSC markers and **(B)** cell cycle-related protein expression was evaluated by flow-cytometry; the mean ± SD (triplicates) percent of stained HSC and **(C)** representative examples are shown; significant differences (p <0.05) by the presence of BM-Str: *, significant differences (p <0.05) by EL4/EL4-v10 exosomes: s; the experiment was repeated 2-times. Expression of cell cycle promoting genes is reduced in HSC cultured on BM-Str, but EL4 exosomes negate the effect of BM-Str on cyclinD1 and c-myc expression.

Settlement of HSC being strongly impaired in the presence of EL4, we focused on the impact of EL4/EL4-v10 on HSC quiescence that is supported by the BM-Str. HSC were seeded on HA or BM-Str and cell cycling was evaluated after 24 h starving and 2 h recovery in the presence of 10% FCS. EL4/EL4-v10 did not interfere with HA-bound HSC cycling. Instead, less HSC were in G2/M when cultured on BM-Str, but were driven efficiently into cell cycle progression in the presence of EL4, but less pronounced in the presence of EL4-v10. Cell cycle progression was also promoted by IM7, but not by K926 (Figure 6C). EL4 exosomes, distinct to EL4-v10 exosomes also promoted HSC/progenitor cell proliferation, when cultured on BM-Str (Figure 6D and F). IM7, and less efficiently K926, interfered with HSC/progenitor cell quiescence (Figure 6E and F). Though after a 2d coculture, CD117, CD135, CD126 and N-cadherin expression was not significantly altered, when HSC were cultured on BM-Str in the presence of EL4 or EL4-v10 exosomes, CXCR4 expression became upregulated in BM-Str cocultures, but was reduced in the presence of EL4 and EL4-v10 (Figure 7A). More impressive changes were seen in signal transduction molecule/transcription factor expression after overnight culture. In line with BM-Str-supported maintenance of HSC quiescence, β-catenin, Lef, cyclinD1 and c-myc expression were low, when HSC were cultured on BM-Str. Expression of cyclinD1 and c-myc became significantly upregulated in the presence of EL4, but not of EL4-v10 (Figure 7B and C).

**Figure 8:**
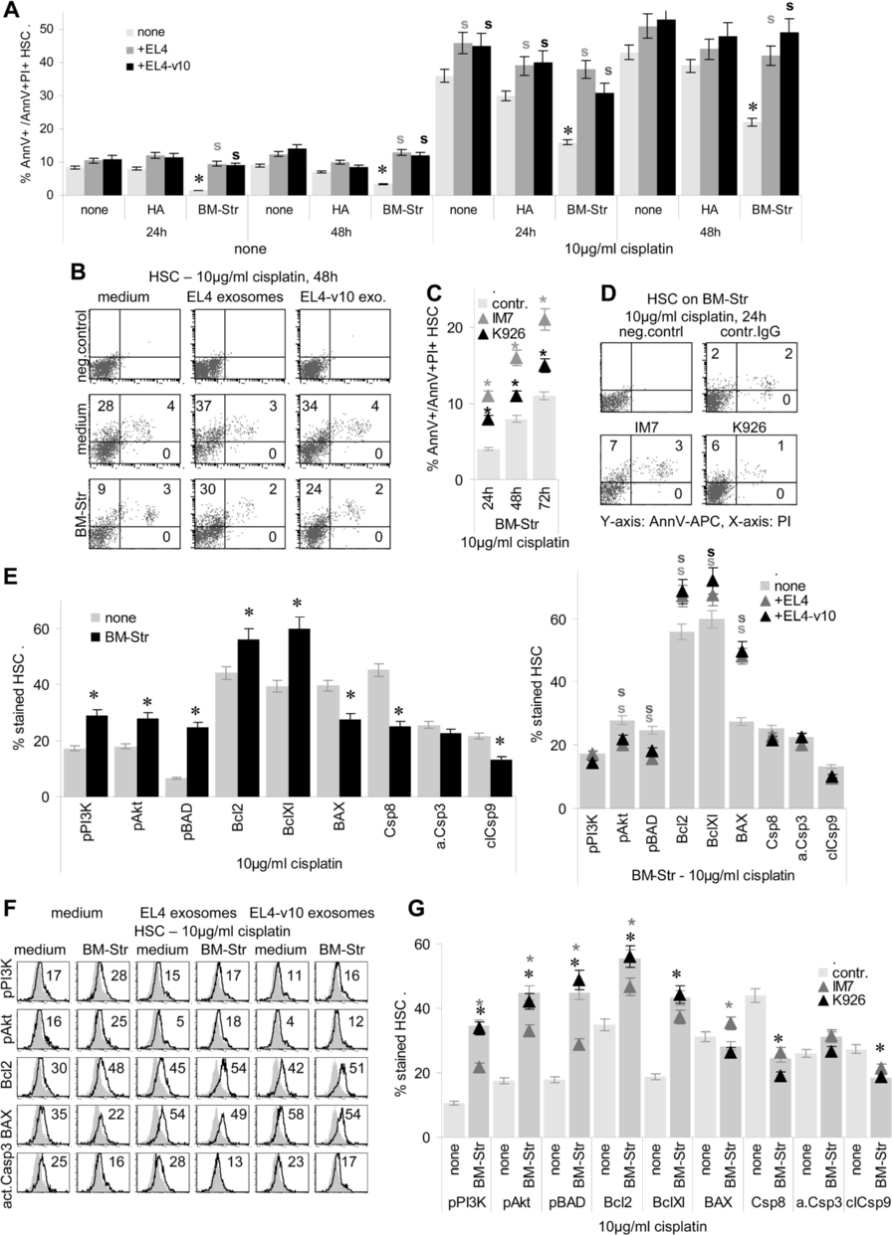
**Impact of EL4 and EL4-v10 on bone marrow stroma-promoted HSC apoptosis resistance. (A-D)** HSC (1×10^6^) were cocultured for 24 h-72 h with mitomycinC-treated EL4/EL4-v10 or EL4/EL4-v10 exosomes on BSA, HA or BM-Str-coated 24-well plates; where indicated, cultures contained 10 μg/ml cisplatin. **(A-D)** Apoptosis of HSC was evaluated by AnnV/PI staining and flow-cytometry (mean ± SD, triplicates and representative examples); **(A)** significant differences (p <0.05) by BM-Str: *; significant differences (p <0.05) in dependence on EL4 or EL4-v10: s; **(C)** significant differences (p <0.05) in the presence of antibody: *. Apoptosis-related protein expression was evaluated by flow-cytometry in HSC cultured overnight in the presence of 10 μg/ml cisplatin in 24-well plates **(E-G)** coated with BSA or BM-Str and **(E,F)** in the presence of EL4 or EL4-v10 exosomes (10 μg/ml) or **(G)** IM7 or K926 (10 μg/ml); **(E-G)** mean percent (triplicates) of stained HSC and representative examples; significant differences (p <0.05) by the presence of BM-Str: *, significant differences (p <0.05) by EL4/EL4-v10: s; significant differences (p <0.05) in the presence of antibody: *. Both EL4 and EL4-v10 interfere with the apoptosis-protective effect of BM-Str, which also is weakly affected by IM7.

Besides supporting HSC quiescence, the BM-Str also contributes to HSC apoptosis resistance [40,41]. EL4 or EL4-v10 interfered with apoptosis resistance of HSC, when protected by HA and more pronounced when protected by BM-Str. This accounted particularly for HSC cultured in the presence of cisplatin (Figure 8A and B). The protective effect of the BM-Str also was largely abolished in the presence of IM7 and was mitigated in the presence of K926 (Figure 8C and D). Low apoptosis of HSC in the presence of BM-Str was accompanied by pronounced PI3K, Akt and BAD phosphorylation, upregulation of Bcl2 and BclXl, and a slight downregulation of BAX. PI3K, Akt and BAD phosphorylation was reduced in the presence of EL4 or EL4-v10. Unexpectedly, expression of Bcl2 and BclXl became upregulated (Figure 8E), which was independent of the presence of BM-Str (data not shown). The latter points towards direct upregulation of anti-apoptotic molecules e.g. via the MAPK or JNK pathway [29,42,43]. EL4 and EL4-v10 did not suffice for caspase activation (Figure 8E and F). Similar to EL4 and EL4-v10, IM7, but not K926 sufficed to inhibit activation of the PI3K anti-apoptotic pathway. IM7 also did not suffice for upregulation of effector caspases (Figure 8G).

Thus, the superior effect of K926 compared to IM7 on survival of mice after i.v. application of EL4-v10 does not rely on K926 more efficiently affecting the leukemic cell, but rather on EL4-v10 not interfering with hematopoiesis that, on the other hand, is affected by EL4 as well as IM7, particularly with respect to HSC quiescence.

Taken together, a blockade of CD44v10 was more efficient than a panCD44 blockade in intra-marrow EL4-v10 growth retardation. The superior effect of K926 compared to IM7 on leukemia growth in the BM mostly is due to EL4-v10 and K926 - distinct to EL4 and IM7 - hardly disturbing the HSC interaction with the BM-Str, which partially negates the impact of anti-panCD44 on leukemia growth.

## Discussion

High CD44 expression in many leukemia and lymphoma has stimulated therapeutic considerations based on a blockade of CD44 that was particularly successful in AML and CML [25,26]. Nonetheless, due to abundant CD44 expression in hematopoietic cells including HSC, anti-panCD44 has also been reported to be burdened by side effects [5,44]. Therefore, we explored for CD44v10 transfected EL4, EL4 not expressing other CD44 variants (M.Zöller unpublished), whether side effects can be circumvented or mitigated by antibodies specific for splice variants that expression is far more restricted [45]. We choose CD44v10 because it is hardly expressed in HSC (M.Zöller, unpublished), but expressed in some leukemia [46], particularly in cutaneous lymphoma [31], Hodgkin’s disease [34] and multiple myeloma [35] and because it is an OPN ligand [unpublished finding], where OPN is important in guiding HSC from the osteogenic to the vascular niche [47-49]. We report that anti-CD44v10 is advantageous as it did not/less severely interfere with HSC embedding and quiescence in the BM-Str niche.

In advance of discussing our findings, it should be remembered that CD44s expression is unaltered in EL4-v10 compared to EL4 cells. This implies that differences between EL4 and EL4-v10 will only be seen as a sequel of genuine functions of CD44v10. Correspondingly, anti-CD44v10 can be expected to confirm genuine CD44v10 activities and in case of inefficacy indirectly strengthens the impact of CD44s.

### Anti-CD44 efficacy in interfering with intra-marrow leukemia growth

Leukemia frequently arising and expanding in the bone marrow, we controlled for the impact of a CD44v-specific antibody on leukemia growth versus hematopoiesis, which was approached by i.v. EL4/EL4-v10 application.

Anti-panCD44 and anti-CD44v10 were not curative, but sufficed for a prolonged survival time. Unexpectedly, therapeutic application of anti-CD44v10 was more efficient than anti-panCD44, although both IM7 and K926 comparably well promoted ADCC [50,51]. In addition, there was no evidence for K926 more efficiently than IM7 driving the leukemia cells into apoptosis. Thus, there remained two alternative, not mutually exclusive explanations. IM7, distinct to K926, affects hematopoiesis and/or EL4/EL4-v10 distinctly interfere with hematopoiesis, a negative impact of the leukemic cells on hematopoiesis not becoming neutralized by IM7.

### EL4/EL4-v10 and the crosstalk with the bone marrow niche

We first controlled on the impact of EL4 and EL4-v10 on BM-Str. EL4 and EL4v10 promoted upregulation of TNFRI, SDF1 and OPN, with EL4-v10 being more efficient in OPN upregulation that could have affected niche egress of EL4/EL4-v10 as well as of HSC [48,52,53]. Upregulated SDF1 might support recruitment of EL4/EL4-v10 as well as of HSC [54-56]. Otherwise, no EL4-/EL4-v10-induced changes in BM-Str were seen that could be expected to affect the crosstalk between EL4/EL4-v10 versus HSC with the stroma.

EL4 adhere more efficiently than EL4-v10 to BM- and spleen-Str, adhesion being significantly inhibited only by IM7, but not by K926. Distinct to adhesion, EL4-v10 had a minor advantage in migration on BM-Str that was neutralized by IM7 and K926. Adhesion and migration remained unaltered, when pretreating the BM-Str with EL4 or EL4-v10 exosomes (data not shown). We briefly want to point out that some unspecific impact of CFSE on functional activity cannot be safely excluded. However, as control settings were treated accordingly, observed differences should be relevant. In line with EL4 more tightly interacting with BM-Str, BM-Str affects EL4 cell cycle progression and proliferation, though to a minor degree and only transiently, the impact of the BM-Str on EL4 being waved in the presence of IM7. Finally, stroma-adherent EL4 and EL4-v10 were protected from a cytotoxic drug. Protection was more pronounced for EL4 than EL4-v10 and was abolished by IM7, but not K926, which findings strengthen our interpretation that EL4 interacts more tightly with the BM-Str than EL4-v10. In mouse models of AML and CML, CD44 targeting suppressed progression and induced differentiation by disrupting the interaction with the bone marrow niche [57], experimental evidence pointing towards apoptosis induction by caspase- or calpain-dependent pathways [58,59] and to inhibition of proliferation through stabilization of the cyclin-dependent kinase inhibitor p27, which resulted in increased association with the cyclinE-Cdk2 complex [58,60].

In the EL4 model, BM-Str protected EL4/EL4-v10 from drug-induced apoptosis and the protective effect was abolished by IM7, confirming a central role for the engagement of CD44. Yet, the thymoma line EL4 obviously does not essentially require the BM-Str for survival, as we observed only a minor effect on EL4/EL4-v10 proliferation. Instead, our findings suggest that EL4, rather than EL4-v10 might compete with HSC for the niche.

### EL4/EL4-v10 competition with HSC for niche embedding

In line with the hypothesis that EL4 competes with HSC for the BM niche was the poorer recovery of HSC in late EL4, but not EL4-v10 TB. Furthermore, transplanted HSC maturation towards T and B cells was more severely impaired in EL4 than EL4-v10 TB and was strongly affected by IM7. Instead, K926 only affected B cell maturation, which is in line with our earlier report on CD44v10 expression in B progenitor cells [30]. In vitro cocultures confirmed that EL4 more efficiently than EL4-v10 competed with HSC for BM-Str adhesion. Furthermore, in the presence of EL4, but less pronounced in the presence of EL4-v10, HSC were driven into cell cycle progression, rare cycling being one of the characteristics of HSC [61]. Cell cycle progression and proliferation of HSC on BM-Str was also promoted by IM7. Corresponding to these findings, only in cocultures with EL4, β-catenin, cyclinD1 and c-myc expression [62-67], which was low in HSC seeded on BM-Str, became upregulated. EL4-v10 exerted no or weaker effects.

Thus, EL4 more strongly than EL4-v10 interfere with the crosstalk between HSC and BM-Str such that HSC are dislodged and possibly differentiate. Though IM7 exerted a similar effect on HSC cocultured with BM-Str, an additive effect of EL4 plus IM7 was not seen or was weak, which suggests that IM7 binds EL4 and HSC with equal efficacy. Alternatively, the crosstalk between both HSC and EL4 with the BM-Str may become affected by stroma-bound IM7 [68,69]. Irrespective of this open question, in concern of the efficacy of IM7 on the survival time of EL4 TB, the benefit of IM7 promoted ADCC will be partly neutralized by the negative impact of EL4 on hematopoiesis. On the other hand, the weaker interaction between EL4-v10 and the BM-Str could explain the higher therapeutic efficacy of K926, where we speculate that weaker adhesion on BM-Str is due to CD44v10 being an OPN ligand and inducing OPN expression (M.Zöller, unpublished). This will favor EL4-v10 motility rather than BM-Str adhesion and will mitigate the impact on the crosstalk between HSC and BM-Str [48,70]. Whether OPN capture by CD44v10 additionally supports HSC quiescence, as OPN can act as a negative regulator of HSC proliferation [48,49,71,72], remains to be explored.

### EL4/EL4-v10 and HSC apoptosis resistance

HA, particularly medium sized HA that was used throughout [73-75] and, far stronger BM-Str protected HSC from cytotoxic drug-induced apoptosis [40,41,76,77]. HSC apoptosis protection was counterregulated by EL4 and EL4-v10 exosomes as well as IM7 and, less pronounced, K926. BM-Str-supported HSC protection was accompanied by activation of the PI3K/Akt pathway [76,78,79], which was not seen in the presence of EL4 or EL4-v10 and became downregulated by anti-panCD44, but not anti-CD44v10. On the other hand, neither EL4/EL4-v10 nor IM7 suffice to initiate caspase9 cleavage or caspase3 activation in the presence of BM-Str. One possible explanation could be that anti-apoptotic triggers, which proceed via distinct pathways [29,42,43], are not affected and suffice to prevent effector caspase activation. In line with this hypothesis was the finding that EL4/EL4-v10 did not promote Bcl2 and BclXl downregulation and that this phenomenon was CD44-independent. Instead, EL4 and EL4-v10 promoted Bcl2 and BclXl upregulation. Thus, it appears that EL4 and EL4-v10 can exert apoptosis promoting as well as protecting effects on BM-Str-embedded HSC, where we speculated that, besides protein interactions, additionally exosomal miRNA might be involved [80,81]. Though the question on the impact of EL4/EL4-v10 and IM7 on the regulation of adult HSC apoptosis resistance, which is still disputed [82,83], requires further elaboration, it is obvious that HSC apoptosis protection is not exclusively regulated via CD44 and also that it is not merely initiated by upregulation of the PI3K/Akt pathway.

## Conclusions

CD44-directed therapeutic approaches have most intensely been explored in hematological malignancies. Using a CD44v10 expressing thymoma, our findings suggest that depending on the type of leukemia, a blockade of CD44v can be preferable. Distinct to CD44s, a blockade of CD44v10 hardly interfered with the crosstalk between HSC and BM-Str, particularly not affecting HSC quiescence. In concern of a concomitant cytotoxic drug therapy, apoptosis resistance of niche-embedded HSC was distorted in the presence of EL4 and, less efficiently, EL4-v10, due to the less tight crosstalk between EL4-v10 with the BM-Str. But, the protective effect of the BM-Str was much weaker for EL4-v10 than EL4. This implies that in combination with cytotoxic drugs, the application of a CD44v-specific antibody could bring about an additional therapeutic advantage.

## Material and methods

### Cell lines

EL4 thymoma cells [84] were transfected with CD44v10 cDNA [85] (EL4-v10). Transfected cells were cloned by limiting dilution in RPMI/10% FCS supplemented with 250 μg/ml G418. EL4-v10 were maintained in RPMI/10% FSC/G418. EL4 were cultured in RPMI/10% FCS.

Antibodies, chemokines, cytokines and matrix proteins are listed in Additional file 1: Table S1 and ref [86].

### Tissue preparation and cell isolation

Mice were bled and sacrificed by cervical dislocation. Femur and tibia, lymph nodes (LN), spleen, thymus and tumor tissue were collected. BM cells (BMC) were flushed with PBS out of the bone, spleen cells (SC) and LN cells (LNC) were obtained by pressing through fine gauze. HSC were enriched by repeated magnetic bead selection, where after depletion of CD4^+^, CD8^+^, CD11b^+^, CD11c^+^, CD16^+^/CD32^+^ and CD19^+^ cells, Ter119^+^ and Gr1^+^ cells were eliminated and finally CD117^+^/SCA1^+^ cells were positively selected. Spleen stroma cells (spleen-Str) and BM-Str, collected from BMC that were depleted of HSC, were cultured for 4 wk in ISCOVES/10% FCS taking care to wash out all non-adherent and loosely adherent cells. When reaching confluence, cells were scrabbed off with a rubber policemen and were split into two flasks. Scrabbed off stroma cells were seeded in 24-well Plates 48 h in advance of starting coculture experiments.

### Exosome preparation

EL4/EL4-v10 were cultured (48 h) in serum-free medium. Cleared supernatants (2×10 min, 500 g, 1×20 min, 2000 g, 1×30 min, 10000 g) were centrifuged (90 min, 100000 g) and washed (PBS, 90 min, 100000 g). The pellet was resuspended and purified by sucrose gradient centrifugation [87].

### Flow cytometry

Cells (1-3×10^5^) were stained according to routine procedures. For intracellular staining, cells were fixed and permeabilized in advance. Apoptosis was evaluated by AnnexinV-FITC or -APC/PI staining. Staining was evaluated using a FACS-Calibur (BD). Analysis was performed by the Cell Quest program. Experiments were repeated at least 3 times.

### Immunohistology

Snap frozen spleen sections (5 μm) were fixed (chloroform/acetone, 1:1, 4 min) and treated with levamisole to ablate tissue alkaline phosphatase activity. Nonspecific binding was blocked using an avidin-biotin-blocking kit (VectorLaboratories, Burlingame, CA, USA), and 2% normal serum derived from the same species as the secondary antibodies. Tissues were incubated with the primary antibodies, washed, exposed to biotinylated secondary antibodies and alkaline phosphatase conjugated avidin-biotin solution and were counterstained with hematoxilin. Images were taken using a Leica DMRBE microscope.

### Adhesion

CFSE labeling was performed in medium without FCS. After washing in medium with 10% FCS, cells were rested for 30 min at 37°C to guarantee CFSE incorporation. Thereafter cells were washed an additional 3-times. CFSE-labeled cells were seeded on BSA (10 μg/ml in bicarbonate buffer, pH 9.6), OPN (2 μg/ml in bicarbonate buffer, pH 9.6), rooster comb HA (mean molecular weight 1–4 million) (100 μg/ml in PBS) or a monolayer of BM-Str in 24-well plates. Plates were coated for 24 h, washed and blocked with 1% BSA in PBS overnight. For BM-Str collection and seeding, see above. After 4 h incubation, non-adherent cells were removed by washing, triplicates of adherent cells were lysed and fluorescence was evaluated photometrically. Adhesion is presented as percent of seeded cells.

### Migration

For videomicroscopy, CFSE-labeled cells (5×10^4^) were seeded on a monolayer of BM-Str-or spleen-Str-coated 24-well plates. Plates were placed under an Olympus IX81 inverse microscope with a Hg/Xe lamp, an incubation chamber (37°C, 5% CO_2_), a CCD camera (Hamamatsu) and a ScanR acquisition soft ware (Olympus, Hamburg, Germany). Two pictures (20-fold magnification)/chamber (2 ms exposure) were taken every 20 min for 12 h. Migration was quantified according to Manual_tracking plugin running in the open-source software Image J. Assays were run in triplicates and tracking was evaluated for 20 individual cells in every well, the presented mean values deriving from 60 individual cells.

### Cell cycle, proliferation and apoptosis

Cells were seeded on BSA-coated plates or on a monolayer of BM-Str. Cell cycle progression was evaluated after starving EL4/EL4-v10 for 48 h and HSC for 24 h, adding RPMI/10% FCS (EL4/EL4-v10) or Iscove'sMEM/10% FCS (HSC) for 2 h. Cells were washed with ice cold PBS and resuspended in 70% cold ethanol and incubated overnight at 4°C. Cells were washed with cold PBS and resuspended in PI-RNAse solution (250U RNAse type I-A, 100 μg/ml, 10 μg/ml PI, =.1% TritonX-100 in PBS). After 15 min incubation at room temperature in the dark, PI staining was evaluated by flow cytometry. For determining cell division, CFSE-labeled cells were starved overnight and cultured for 24 h-72 h, determining CFSE-dilution by flow-cytometry. Apoptosis was determined by AnnV (1 μg/ml)/PI (0.3 μg/ml) staining. Where indicated, cultures contained 2.5-20 μg/ml cisplatin. Mean values ± SD of triplicates are presented.

### Cytotoxicity assay

ADCC activity was evaluated after *in vitro* restimulation in the presence of 100 U/ml IL2 for 48 h. Where indicated, cultures contained 10 μg/ml anti-CD44 (IM7) or anti-CD44v10 (K926). ^3^H-thymidine labeled (12 h, 10 μCi/ml ^3^H-thymidine) target cells (10^4^/well) were seeded on titrated numbers (10×10^5^ - 6×10^4^) of effector cells in 96 well plates. After 6 h at 37°C, plates were harvested, and radioactivity was determined in a β-counter. Cytotoxicity is presented as % cytotoxicity = 100 × (counts in control wells - counts in test wells)/(counts in control wells). The spontaneous release [100 × (total counts – counts in control wells)/total counts)] of the target cells ranged between 6%-10%. Mean values ± SD of triplicates are presented. SD were in the range of 3%-5%.

### Animal experiments

C57BL6 mice (5/group) received an intravenous (i.v.) injection of 1×10^6^ EL4 or EL4-v10 and 2×/week 100 μg/mouse IM7 or K926 in 200 μl 0.9% NaCl solution. Mice were sacrificed according to weight loss and fatigue. To evaluate tumor cell homing, mice (3 mice/group) received 1×10^7^ CFSE-labeled tumor cells, which were preincubated (30 min, 4°C) with 200 μg IM7 or K296 in 200 μl 0.9% NaCl solution (i.v.). Mice were sacrificed after 48 h and 72 h. Organs were excised and single cell suspensions were prepared. The presence of CFSE-labeled tumor cells was evaluated by flow cytometry. For evaluating the impact of EL4/EL4-v10 and IM7/K926 on HSC maturation BALB/c SCID mice (5/group) received an i.v. injection of 2×10^6^ C57BL6 HSC. Where indicated, mice received concomitantly 1×10^6^ EL4 or EL4-v10, i.v. and 1 day later IM7 or K926 (100 μg / mouse in 100 μl 0.9% NaCl solution). The antibody application was repeated every 5th day. Mice were sacrificed after 3 wk. BM, thymus, LN and spleen were excised and the recovery of donor cells (H-2^b^), B cells CD4^+^, CD8^+^ and CD4^+^CD8^+^ T cells as well as of SCA1^+^ and CD34^+^ was evaluated by flow cytometry. The mean percent of marker-positive cells per organ from 5 mice is presented. Animal experiments were approved by the local Government.

### Statistics

Significance was evaluated by the two tailed Student’s t-test (in vitro assays) or the Kruskal-Wallis test (in vivo assays). P values <0.05 were considered significant and are indicated by an asterisk or s.

## Abbreviations

ADCC: Antibody-dependent cellular cytotoxicity; BM: Bone marrow; BMC: BM cells; CD44s: CD44 standard isoform; CD44v: CD44 variant isoform; EL4-v10: CD44v10 transfected EL4 cells; HA: Hyaluronic acid; HSC: Hematopoietic stem cells; i.v: Intravenous; LIC: Leukemia initiating cells; LN: Lymph node; LNC: LN cells; Mϕ: Macrophage; NK: NK cells; OPN: Osteopontin; SC: Spleen cells; spl: Spleen; Str: Stroma; TB: Tumor-bearer.

## Competing interests

None of the authors has a conflict of interests.

## Authors’ contributions

UE performed and analyzed experiments and critically revised the drafting of the manuscript, APM performed and analyzed experiments, XG performed experiments, MWB critically revised the drafting of the manuscript, MZ designed the study, performed and analyzed experiments and wrote the manuscript. All authors read and approved the final manuscript.

## Supplementary Material

Additional file 1 CD44 standard and CD44v10 isoform expression on leukemia cells distinctly influences niche embedding of hematopoietic stem cells. Click here for file

## Supportive information

1 Table listing antibodies and other reagents.
