# Supplementary material for: CD44 standard and CD44v10 isoform expression on leukemia cells distinctly influences niche embedding of hematopoietic stem cells
Source: J Hematol Oncol. 2014 Mar 31;7:29. doi: 10.1186/1756-8722-7-29 (PMC4022365; doi:10.1186/1756-8722-7-29)
Supplement: Additional file 1 — CD44 standard and CD44v10 isoform expression on leukemia cells distinctly influences niche embedding of hematopoietic stem cells. [file 1756-8722-7-29-S1.pdf]

Additional file

CD44 standard and CD44v10 isoform expression on leukemia cells distinctly influences niche embedding of hematopoietic stem cells

Ulrike Erb[1], Amelie Pajip Megaptche[1], Xiaoyu Gu[1], Markus W. Büchler[2], Margot Zöller[1]

[1]Department of Tumor Cell Biology, University Hospital of Surgery, [2]University Hospital of Surgery, Heidelberg, Germany

Table 1

Table 1A

Antibodies

**Antibody**	**species**	**supplier**
Akt / p-Akt	rabbit	BD[1], HD, G
BAD / p-BAD	rabbit	BD, HD, G
BAX	rabbit	BD, HD, G
Bcl2	rabbit	BD, HD, G
BclXl	rabbit	BD, HD, G
ß-catenin	rabbit	BD, HD, G
bFGF	mouse	BD, HD, G
act.Caspase3	rabbit	BD, HD, G
Caspase 8	rabbit	BD, HD, G
cleaved Caspase9	rabbit	Cell Signaling, Frankfurt, G
CCL5 (Rantes)	rabbit	Biotrend, Cologne, G
CCR4	rabbit	R&D, Wiesbaden, G
CCR9	rat	R&D, Wiesbaden, G
CD9	rat	BD, HD, G
CD11b (YBM6.610)	rat	EACC[2]
CD14	rat	BD, HD, G
CD16	rat	BD, HD, G
CD32 (2.4G2)	rat	EACC
CD34	rat	BD, HD, G
CD44 (IM7)	rat	EACC
CD44v10 (K926)	rat	[31]
CD45R (B220)	rat	EACC
CD54 (YN171.7.4)	rat	EACC
CD63	rabbit	SC[3], HD, G
CD80	rat	BD, HD, G
CD81	rat	BD, HD, G
CD86	rat	BD, HD, G
CD90 (YTS154)	rat	EACC
CD105	rat	BD, HD, G
CD106	rat	BD, HD, G
CD117	rat	BD, HD, G
CD135	rabbit	SC, HD, G
c-myc (9E10)	mouse	EACC
CXCL10 (IP10)	rabbit	Biotrend, Cologne, G
CXCL12 (SDF1)	rabbit	SC, HD, G
CXCR3 (CD182, IP10-R)	rat	SC, HD, G
CXCR4 (CD184, SDF1-R)	rabbit	SC, HD, G
cyclinD1	rabbit	Dianova, Hamburg, G
Gr1	rat	BD, HD, G
H-2^b (K7-65)	mouse	ref.87
H-2^d (K9-18)	mouse	ref.87
HSP70	rabbit	BD, HD, G
I-A^b (K25-137)	mouse	ref.87
IFNγ-R (CD119)	rat	BD, HD, G
IgM	rat	BD, HD, G
IL6	rat	BD, HD, G
IL6Ra (CD126)	rat	BD, HD, G
IL10R (CD210)	rat	Biolgegend, Uithoom,NE
Lef	rabbit	SC, HD, G
MFGE8	rabbit	SC, HD, G

Table 1A continued

NK (PK136)	rat	EACC
OPN	rabbit	R&D, Wiesbaden, G
PI3K / pPI3K	rabbit	SC, HD, G
SCA1 (E13.161)	rat	EACC
Ter119	rat	BD, HD, G
TNFα	hamster	BD, HD, G
TNFRI (CD120a)	hamster	BD, HD, G
TNFRII (CD120b)	hamster	BD, HD, G

Table 1B

Matrix proteins, Cytokines / Chemokines, Chemicals

**Substance**	**Supplier**	**Dose**
AnnexinV-APC, -FITC, -PE	BD, HD, G	variable
BSA	Sigma, Munich, G	100µg/ml
Hyaluronic acid	Sigma, Munich, G	100µg/ml
IL2	IT[4], Friesoythe, G	3µg/ml
OPN	IT, Friesoythe, G	2µg/ml
mitomycin	Sigma, Munich, G	100µg/ml
CFSE	Invitrogen, Karlsruhe, G	0.5-2µM
cisplatin	Sigma, Munich, G	1-40µg/ml
PI	IT, Friesoythe, G	0.08ng/ml

[1] BD: Becton Dickinson, Heidelberg, German, [2] EACC: European Animal Cell Culture Collection, Porton Down, UK, [3] SC: Santa Cruz, Heidelberg, Germany, [4] Immunotools, Friesoythe, Germany
